# A method to evaluate enhanced rock weathering using intact soil monoliths under field conditions

**DOI:** 10.1016/j.mex.2024.102971

**Published:** 2024-09-23

**Authors:** Caio F. Zani, Arlete S. Barneze, Gerlinde B. De Deyn, J. Frans Bakker, Kevin Stott, David A.C. Manning

**Affiliations:** a School of Natural and Environmental Sciences, Newcastle University, Kings Road, Newcastle upon Tyne, England NE1 7RU, United Kingdom; b UK Centre for Ecology & Hydrology, Lancaster Environment Centre, Library Avenue, Bailrigg, Lancaster, England LA1 4AP, United Kingdom; c Soil Biology Group, Wageningen University & Research, Droevendaalsesteeg 3, 6708 PB Wageningen, the Netherlands; d Unifarm, Wageningen University & Research, Bornsesteeg 48, 6708 PE Wageningen, the Netherlands; e Geography and Environmental Sciences, Faculty of Engineering and Environment, Northumbria University, Newcastle upon Tyne, England NE1 8ST, United Kingdom

**Keywords:** Carbon cycling, Measurement, Monitoring, Nutrient cycling, Rockdust, Soil leachates, Verification, Water balance, Intact soil monolith for enhanced rock weathering evaluation under field conditions

## Abstract

Enhanced rock weathering (ERW) has attracted considerable attention as a carbon dioxide removal (CDR) strategy. However, a reliable method for accurately measuring, monitoring, and verifying carbon dioxide (CO_2_) removal, particularly under field conditions, remains elusive. Here we describe a method for installing soil monoliths in an in situ buried apparatus that allows collection of water draining through a soil, undisturbed by external environmental factors that may affect similar apparatus located above ground. The method provides a robust, cost-effective means of collecting, developing, and establishing soil monoliths, allowing through drainage soil water sample collection and analysis, and so facilitating estimation of ERW CO_2_ removal. A 200 mm diameter polyvinyl chloride (PVC) pipe is inserted into the soil to extract intact monoliths from a site of interest, withdrawn and then fitted with a basal double socket coupling and end cap for leachate collection. It is buried to reproduce soil environmental conditions, and water is collected via a sampling tube to surface. Validity was confirmed through an experimental trial with 36 monoliths over 6 months. This method enables accurate chemical analysis of solute draining through the soil monolith, which can be used to validate models of ERW efficacy.

• PVC pipes are inserted into the target soil and subsequently extracted to retrieve intact soil monoliths
• PVC sockets, equipped with a mesh and a geotextile membrane in the middle to retain the collected intact soil monolith and prevent soil particle transport, are then attached to the PVC pipe
• PVC caps, featuring a small drainage tube attached to its outer side, are used to collect the leachate at the bottom part of the system.

## Graphical abstract

**Figure:**
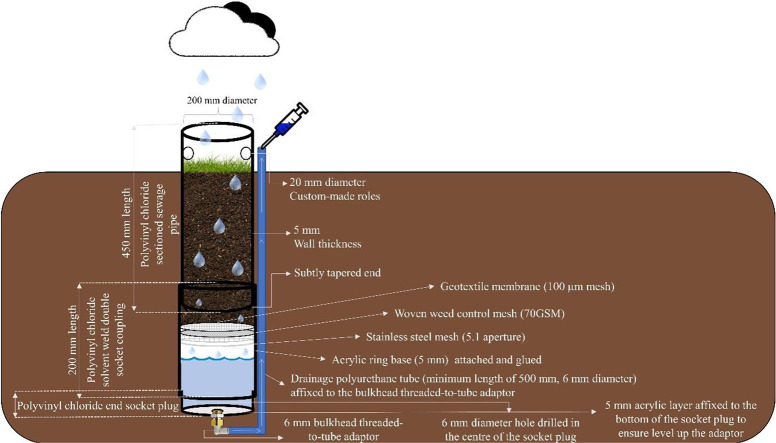

Specifications table

**Table:** 

Subject area:	Environmental Science
More specific subject area:	Enhanced rock weathering (ERW)
Name of your method:	Intact soil monolith for enhanced rock weathering evaluation under field conditions
Name and reference of original method:	The method described here was based on the “Plant communities can attenuate flooding induced N_2_O fluxes by altering nitrogen cycling microbial communities and plant nitrogen uptake” [1] methodology, after adaptation for specific evaluation of enhanced rock weathering potential and effects.
Resource availability:	Reagents and equipment are listed in the Materials section.

## Background

Enhanced rock weathering (ERW) is increasing as an ambitious and scalable strategy for atmospheric carbon dioxide (CO_2_) removal [2]. This natural process, which occurs over geological timescales, involves the reaction of silicate minerals with atmospheric CO_2_ and water to form bicarbonates and, eventually, stable carbonate minerals that might store carbon (C) for millennia. By applying finely ground silicate rocks, such as basalt to soils, ERW aims to enhance this process and simultaneously provide co-benefits such as soil fertility improvement and mitigation of ocean acidification [3].

Previous studies have demonstrated the potential for ERW for atmospheric C removal in a controlled setting such as in greenhouses or laboratories [4,5]. For example, Kelland et al. [4] used mesocosm experiments to explore the application of basalt rock dust in greenhouse conditions, finding increased soil pH, nutrient availability, and crop yields alongside CO_2_ sequestration. The model used by Kelland et al. [4] is widely used and calibrated against solution compositions from experiments of this type. Similarly, Vienne et al. [5] conducted mesocosm experiments to evaluate the co-benefits and potential risks associated with basalt application, highlighting its dual role in C capture and soil health enhancement. However, these controlled studies often fail to account for the complex interactions and variables present in field conditions, such as varying weather patterns, soil types, and biological activity. In view of this, in an attempt to address variable weather conditions, Buckingham et al. [6] assessed ERW efficacy using “tubes on a roof” method with coarse basalt dust. While this study has provided some valuable findings, a more recent study [7] is critical, highlighting the need for a more robust approach, which could deliver more reliable and accurate measurement, monitoring, and verification of EW efficacy studies, especially under field conditions.

To address this gap, we developed an innovative method that involves a field experiment using intact soil monoliths within a buried mesocosm system, which is a significant departure from the typical greenhouse or laboratory settings. This approach aims to address the challenges associated with maintaining soil environmental conditions in both laboratory, greenhouse and/or above-ground mesocosm experiments. The method is based on the approach reported by Barneze et al. [1], including the development of components, collection, and establishment of intact soil monoliths in the field, which offers a precise and reliable means to measure compositional changes in the soil solution over time under real-world conditions. This is done by allowing water to drain through soil monoliths with different treatments under appropriate field conditions and then collecting water samples for chemical analysis. Despite being enclosed in polyvinyl chloride (PVC) pipe, the intact soil monoliths remain influenced by the surrounding environmental conditions and are consequently subject to natural field effects, such as variations in soil moisture and temperature. This is particularly significant for soil water cycling processes, which we highlight as a vital component of ERW assessment. Therefore, in the context of ERW, this approach facilitates precise measurement of changes in soil solution chemical composition that can be used to model CO_2_ removal, and provides a more accurate reflection of field conditions for reliable and applicable measurement, monitoring, and verification procedures.

## Method details

**Fig. 1:**
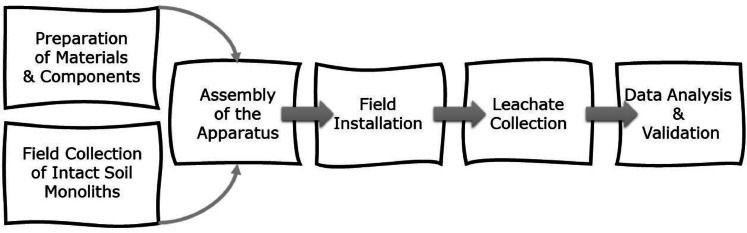
Workflow diagram illustrating the sequential steps involved in the methodological process.

The approach described below is based on modification of the approach reported by Barneze et al. [1], beginning with 1) the development of the components, 2) collection of the intact soil monoliths, 3) connection of the components (assembly), 4) the establishment of intact soil monoliths in the field, 5) leachate collection and concluding with 6) data analysis and validation (Fig. 1).

In a nutshell, the method begins with a workshop phase, which involves the acquisition and preparation of materials, including PVC pipes, double socket couplings with mesh and geotextile membrane, and socket plugs with drainage tubes for leachate collection. Customisation of the PVC pipes follows such as drilling holes and bevelling edges. Next is the fieldwork phase, which includes the collection of intact soil monoliths and assembly of the apparatus. The double socket coupling with mesh is attached to the bottom of the PVC pipe to secure the soil monolith, and the socket plug with drainage tube is connected at the base of the system. The field installation involves digging trenches or cylindrical holes in the target field using manual or mechanical tools, inserting the soil monolith system, and backfilling soil around the pipe, ensuring correct placement of the sampling tube. Leachate collection then proceeds for the duration of interest, where water leaching through the soil monoliths is collected via the sampling tube. In the final phase, data analysis and validation, the collected leachate is analysed for chemical components (e.g., bicarbonate, calcium, magnesium), and the data is used to measure solute flux and assess the efficacy of ERW.

**Fig. 2:**
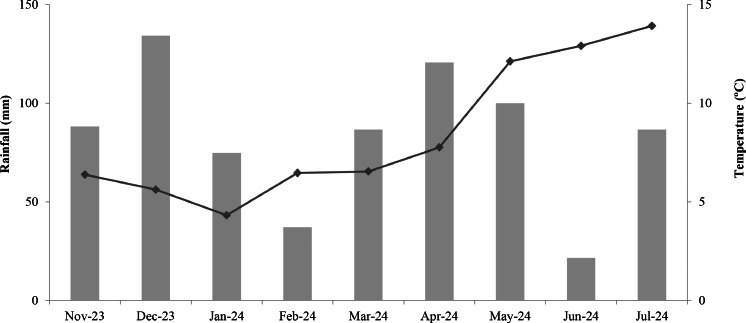
Average monthly air temperature (line) and rainfall (bars) at Cockle Park farm, Northumberland, Northeast England, UK, between November 2023 and July 2024.

The ability of the apparatus to deliver water samples for chemical analysis has been tested in an experimental study of the weathering of basaltic rock and biochar under field conditions (ongoing experiment) and the steps aforementioned are fully described below. The experiment is located at Newcastle University's Cockle Park Farm, Northumberland, UK (Latitude: 55.2144°, N Longitude: −1.6854° W) and consists of 36 monoliths in a completely randomised block design. The site experiences a marine west coast climatic, with an average temperature and total precipitation during the course of the experiment (6 months – from November 2023 to April 2024) of 8.4 °C and 749.8 mm, respectively, with a maximum temperature of 25 °C and a minimum of −4 °C recorded (Fig. 2, data from an on-site automated weather station).

All the intact monoliths were collected from a Royal Air Force (RAF) base located in Leeming, North Yorkshire, UK, (Latitude: 54.2865° N, Longitude: −1.5168° W). Following construction of the apparatus at Cockle Park Farm, they received the following treatments: control, basalt (equivalent to 20 t ha^-1^), biochar (5 t ha^-1^), and basalt + biochar (20 t ha^-1^ + 5 t ha^-1^). These treatments were selected to examine the effects of basalt and biochar, as well as their combined effects, allowing an assessment of the potential removal of atmospheric CO_2_ as both inorganic and organic C. It is important to highlight that the results presented here are simply intended to demonstrate the ability of the new apparatus to deliver samples of the solution draining through the soil for chemical analysis. The experiment is still ongoing, and this publication does not intend to discuss the findings in detail (this is the aim of another publication currently in preparation). However, the results presented here serve as a sound basis for the validation of the method proposed and are therefore briefly outlined in the Method Validation section.

The intact monoliths used in the experiment were carefully collected considering the same soil type, Cambisol (WRB, 2015), which is characterised by freely draining, slightly acid loamy soil that is naturally low in fertility, and in this case, with above-ground vegetation (perennial ryegrass). Specifically, soil analysis for the 30 cm depth indicated an average soil organic C content of ∼2 %, soil inorganic C content of ∼0.2 %, pH of 6.3, and particle-size distribution with an average of 36.5, 40.9, and 22.2 % of sand, silt and clay, respectively (loam soil).

**Fig. 3:**
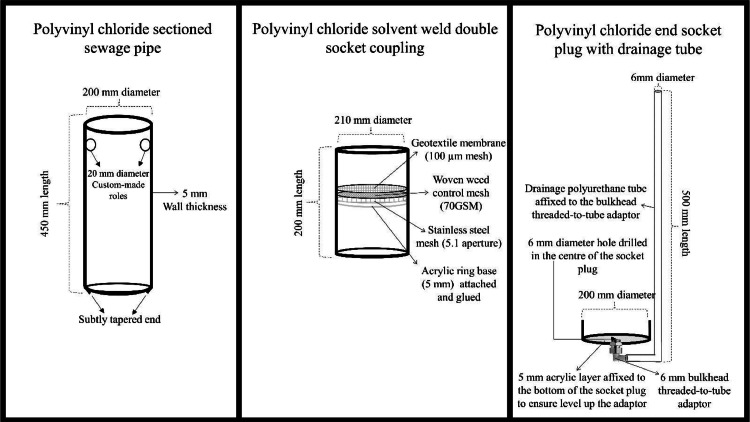
Schematic overview of the parts of the intact soil monoliths system for field conditions to evaluate enhanced rock weathering carbon removal.

(1) Development of the components. The apparatus consists of a polyvinyl chloride (PVC) pipe, a PVC solvent-welded double socket coupling (double socket for short, where the water leachates are stored), and a PVC end socket plug (cap for short) (Fig. 3).

All of these PVC parts are standard for the installation of household plumbing and are readily available from builders’ merchants. In this case, they were acquired from Plastics Express, UK (www.plastics-express.co.uk).

**Fig. 4:**
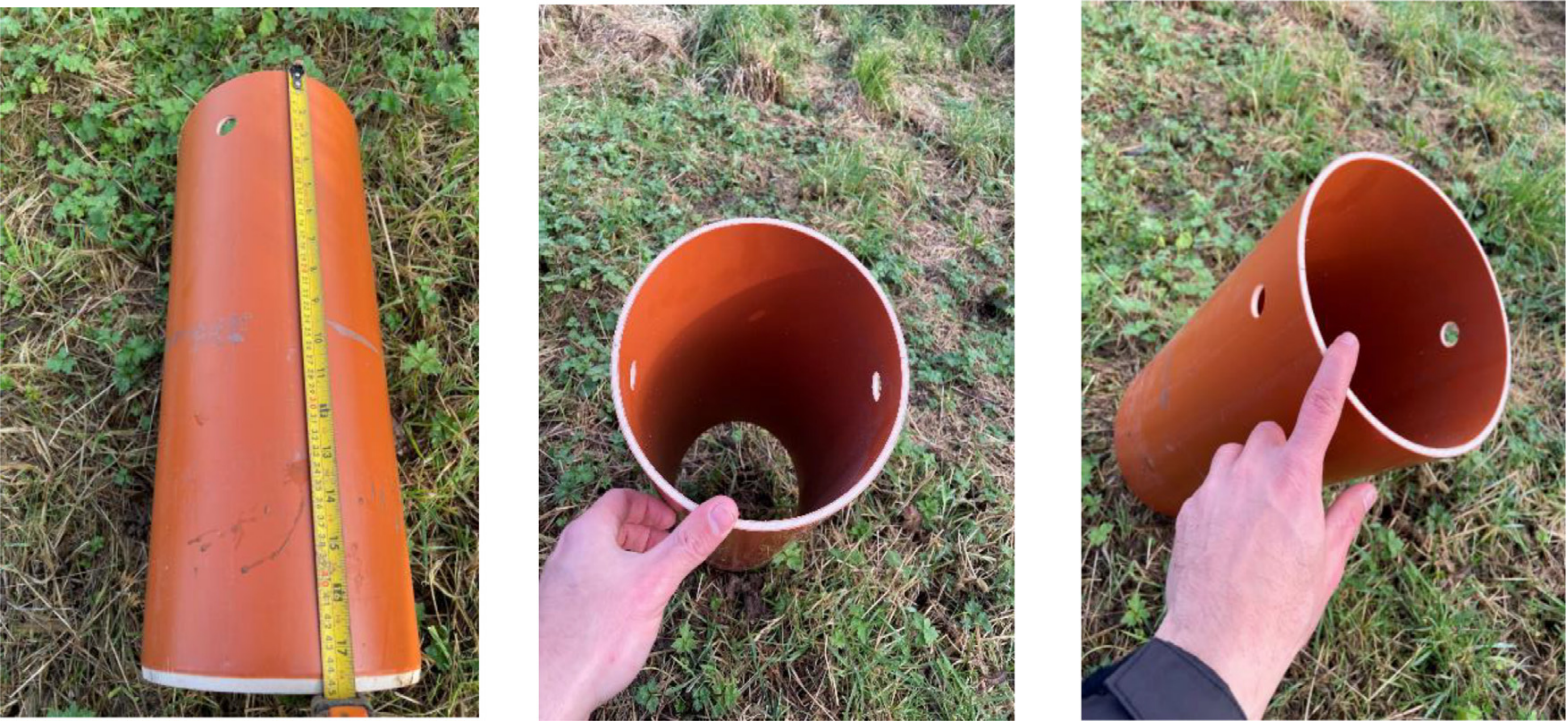
PVC pipe with two custom-made holes at one end and bevelled opposite end.
(b) Double socket: In the middle of the double socket (200 mm in diameter and length, wall thickness of 5 mm), an acrylic ring base (5 mm, Kitronik, UK; kitronik.co.uk) was attached and glued, followed by a stainless steel mesh (grade 304, 5.1 mm aperture, Mesh Direct, UK; www.meshdirect.co.uk), a woven weed control mesh (70GSM, Jardim Perfect Garden, UK), and a geotextile membrane (Spudlica 100 gsm non-woven fleece fabric, 100 µm mesh size). These components were incorporated into the middle of the double socket to secure the soil core, preventing it from sliding through the pipe and preventing particle migration into the collected leachate (Fig. 5).

(a) PVC pipe: 200 mm pipes, with a wall thickness of 5 mm, were cut into segments 450 mm long. Two custom-made holes, each with a diameter of 20 mm, were created with a spacing of approximately 25 mm from one end of the pipe, while the opposite end of the pipe was slightly bevelled, to create a cutting edge (Fig. 4).

**Fig. 5:**
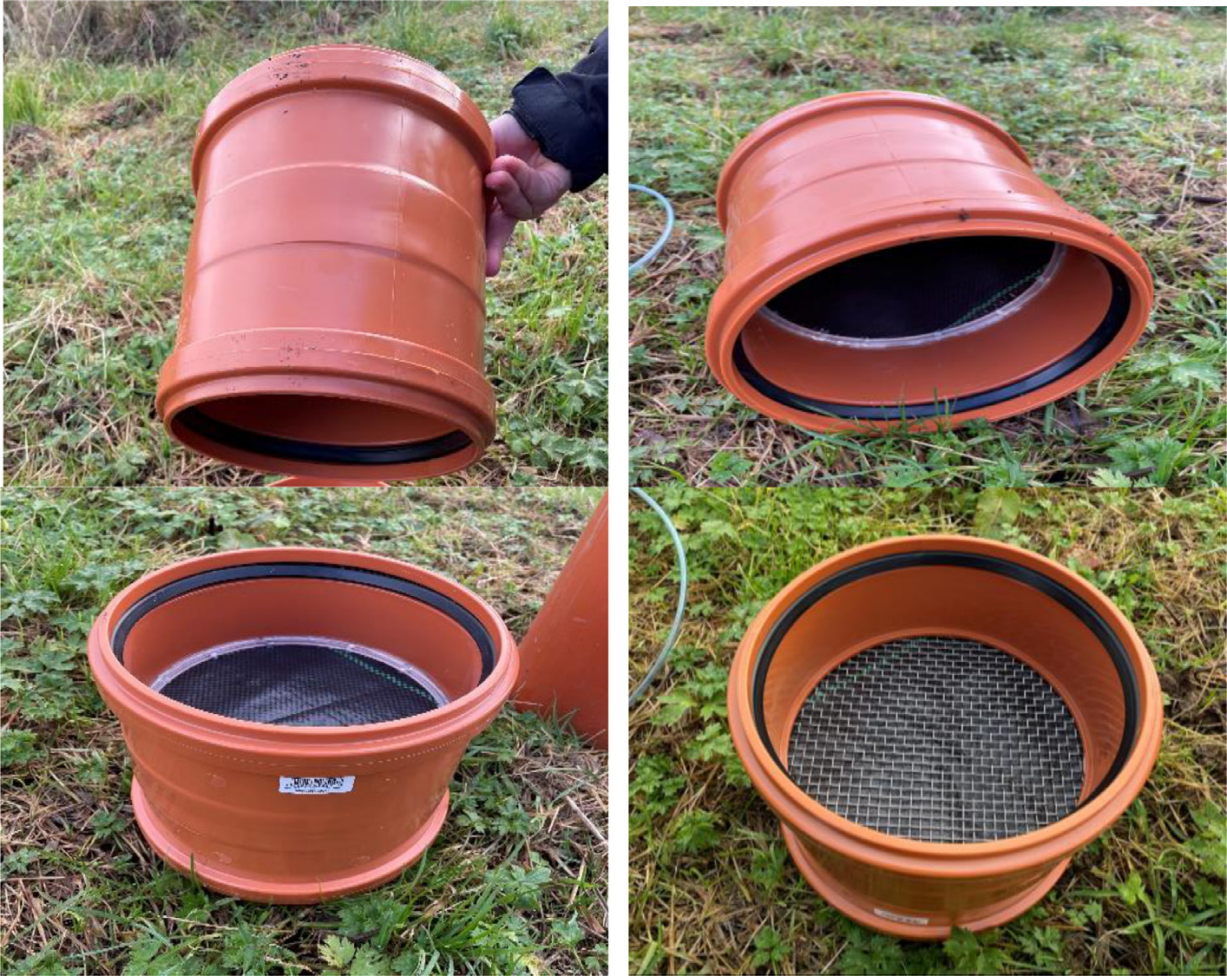
PVC solvent welded double socket coupling with acrylic ring base, a woven weed control mesh and a geotextile membrane in the middle.
(c) Socket plug: A 6 mm diameter hole was drilled in the centre of the socket plug, and a bulkhead threaded-to-tube adaptor (6 mm, RS, UK; www.rs-components.com) was inserted and connected. Additionally, an acrylic layer (5 mm, Kitronik, UK) was fixed to the bottom of the socket plug to ensure the adaptor was level, preventing water from pooling on one side of the plug (Fig. 6).

**Fig. 6:**
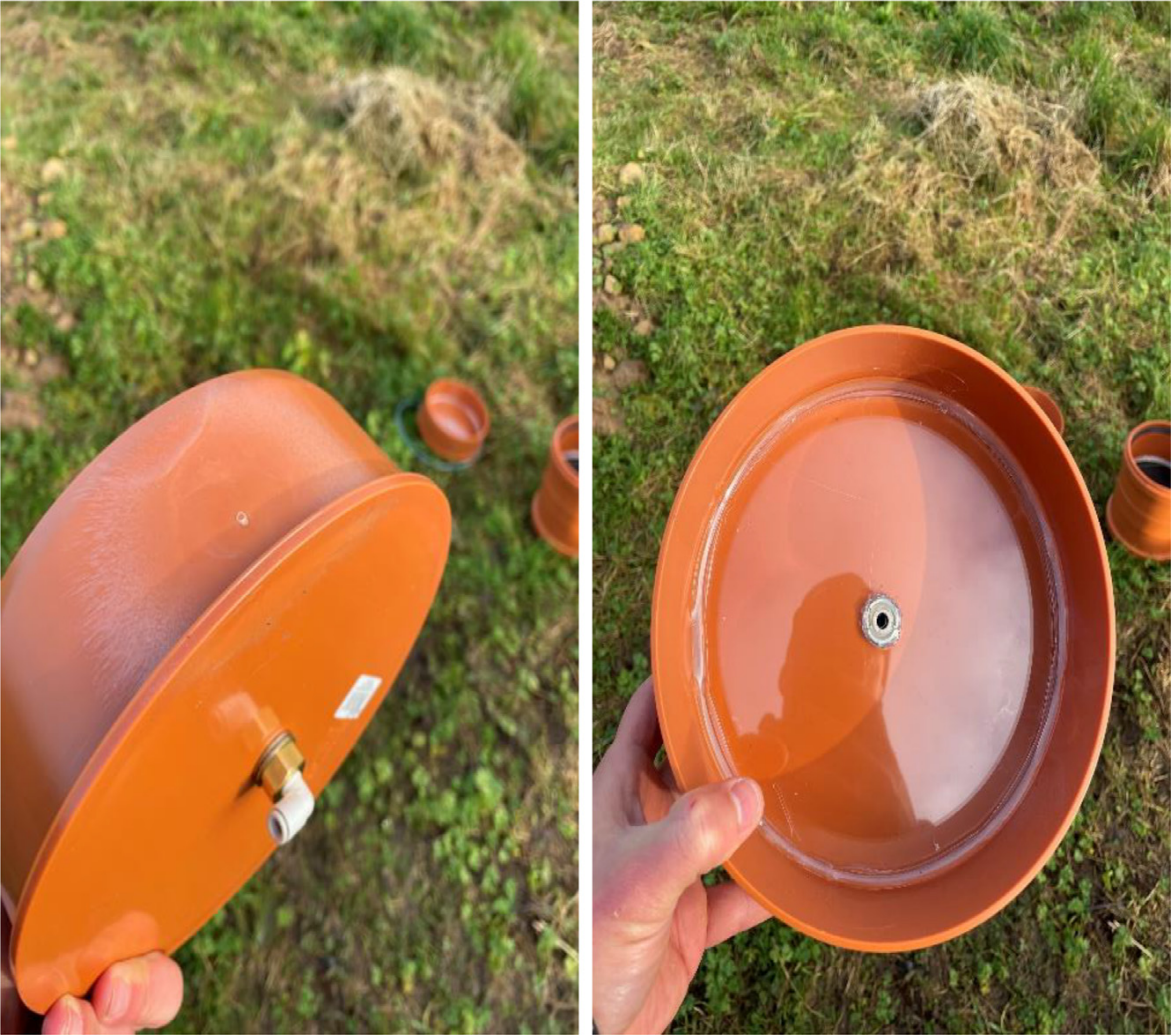
Polyvinyl chloride end socket plug with an acrylic layer on the inner bottom surface for levelling purposes.

**Fig. 7:**
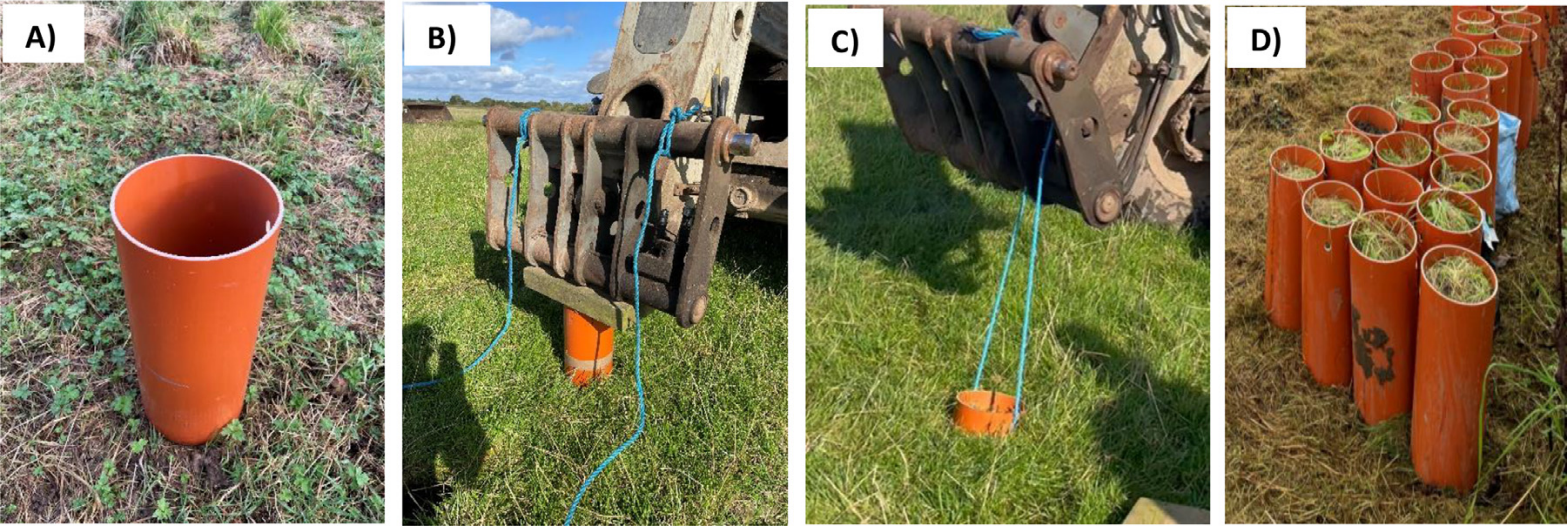
Polyvinyl chloride sectioned pipe positioned with the two custom-made holes oriented upward and slightly taped end in contact with the soil surface (A), a wooden block placed on top and a tractor-mounted front loader pushing it into the ground (B), a polypropylene rope threaded through the holes and connected to the front loader for the extraction of the intact soil monolith (C), and intact soil monoliths extracted using the sectioned pipe (D).
(b) A wooden block is placed on the sectioned PVC pipe which is then pushed into the soil using a front loader mounted on a tractor until it reaches the level of the two custom-made holes, so the soil surface remains approximately 25 mm from the upper end of the pipe (Fig. 7B).
(c) A polypropylene rope (6 mm diameter) is threaded through the holes and connected to the front loader to extract the intact soil monolith from the ground (Fig. 7C). The pipe now contains soil in an undisturbed condition (Fig. 7D).

**Fig. 8:**
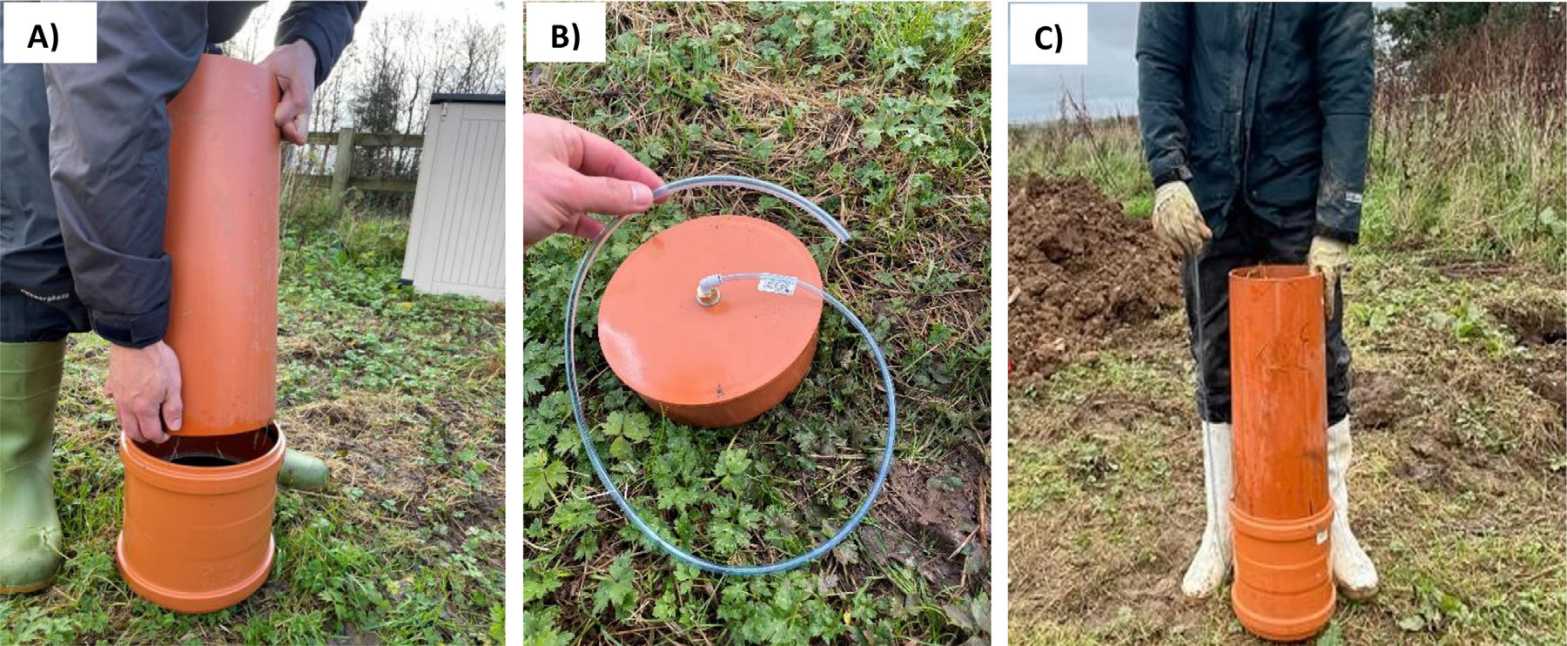
A double socket coupling being positioned at the tapered end of the PVC pipe containing the undisturbed soil monolith (A), sampling tube attached to the bulkhead threaded-to-tube adaptor via the socket plug (B), and the entire system completed and sealed (C).
(b) The socket plug, with a central 6 mm diameter threaded-to-tube adaptor, is attached to the opposite end of the double socket coupling, thereby sealing the system.
(c) For fluid sampling, a polyurethane tube (minimum length of 500 mm, 6 m diameter, RS, UK) is fixed to the bulkhead threaded-to-tube adaptor (Fig. 8B), completing the entire system (Fig. 8C).

**Fig. 9:**
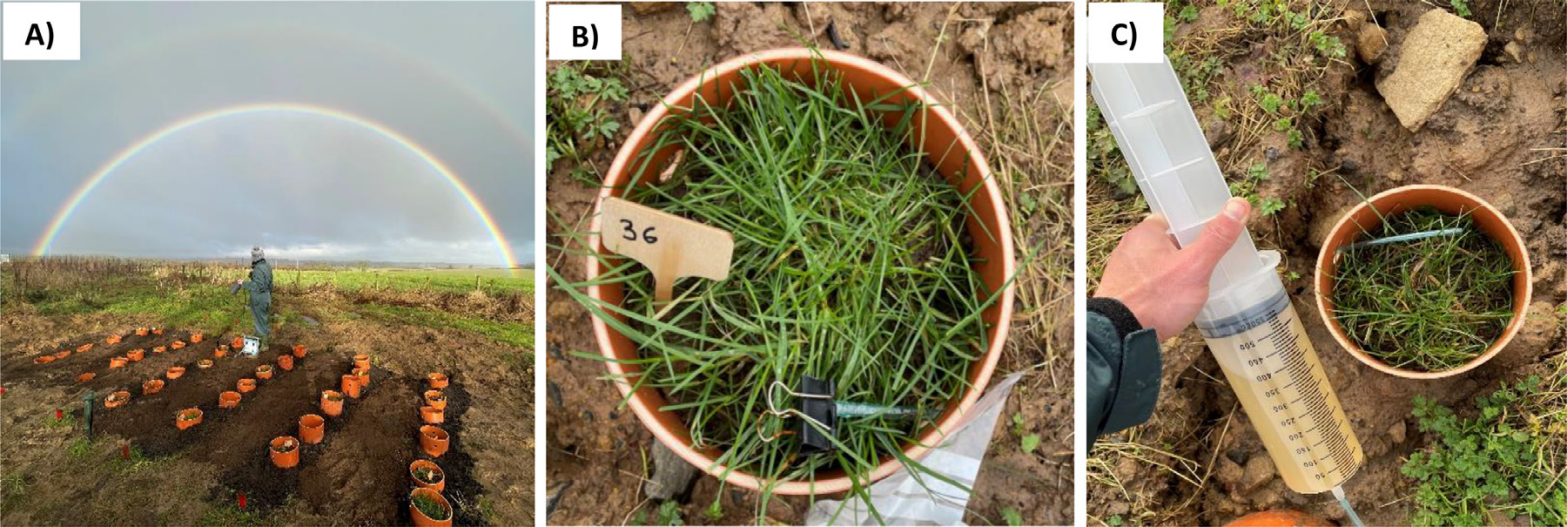
Field experimental area of enhanced rock weathering utilising intact soil monolith systems at Newcastle University's Cockle Park Farm (A), a close-up image of an intact soil monolith backfilled into the trench with the sampling tube positioned outside the trench (B), and collection of leached water via the sampling tube (C).

(2) Collection of the intact soil monoliths in the field.

(a) The sectioned PVC pipe (450 mm in length) is positioned on the desired field plots, ensuring that the two custom-made holes are oriented upward, with the bevelled end in contact with the soil surface (Fig. 7A).

(3) Connecting the components.

(a) After retrieving the intact soil monolith, a double socket coupling is positioned at the bevelled end of the PVC pipe containing the undisturbed soil, ensuring that the geotextile membrane is in direct contact with the bottom part of the intact soil monolith and the acrylic ring base is securely fastened to prevent the monolith from sliding through the pipe (Fig. 8A).

(4) Establishment of intact soil monoliths in the field.

(a) A tractor or other equipment (such as a manual or mechanical post-hole borer or drill) can be employed to excavate trenches (or cylindrical holes) with a depth of 400 mm and a width of 200 mm.
(b) The intact soil monolith with the system is subsequently positioned within the excavated trench, and soil is backfilled around its perimeter (Fig. 9A). It is necessary to ensure that approximately 50 mm of the PVC pipe protrudes above the soil surface and that the collection part of the sampling tube is positioned to enable the leached water to be sampled (Fig. 9B and C).

## Method validation

**Fig. 10:**
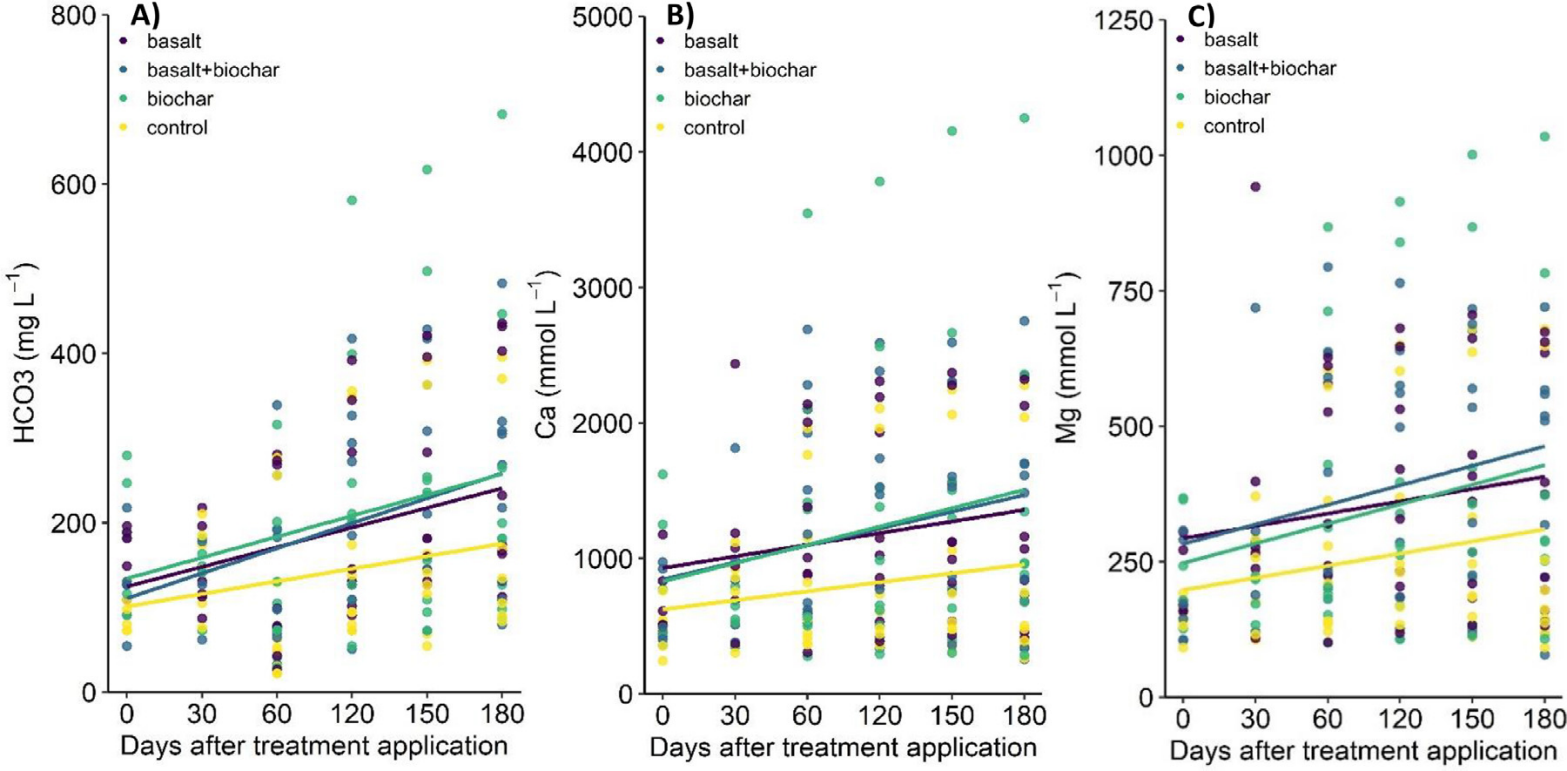
Temporal variations in the concentrations of bicarbonate (HCO_3_^-^) in mg l^-1^ (A), calcium (Ca^2+^) in mmol l^-1^ (B), and magnesium (Mg^2+^) in mmol l^-1^ (C) in leachates collected following basalt and/or biochar treatment application and unamended soil as control.

**Fig. 11:**
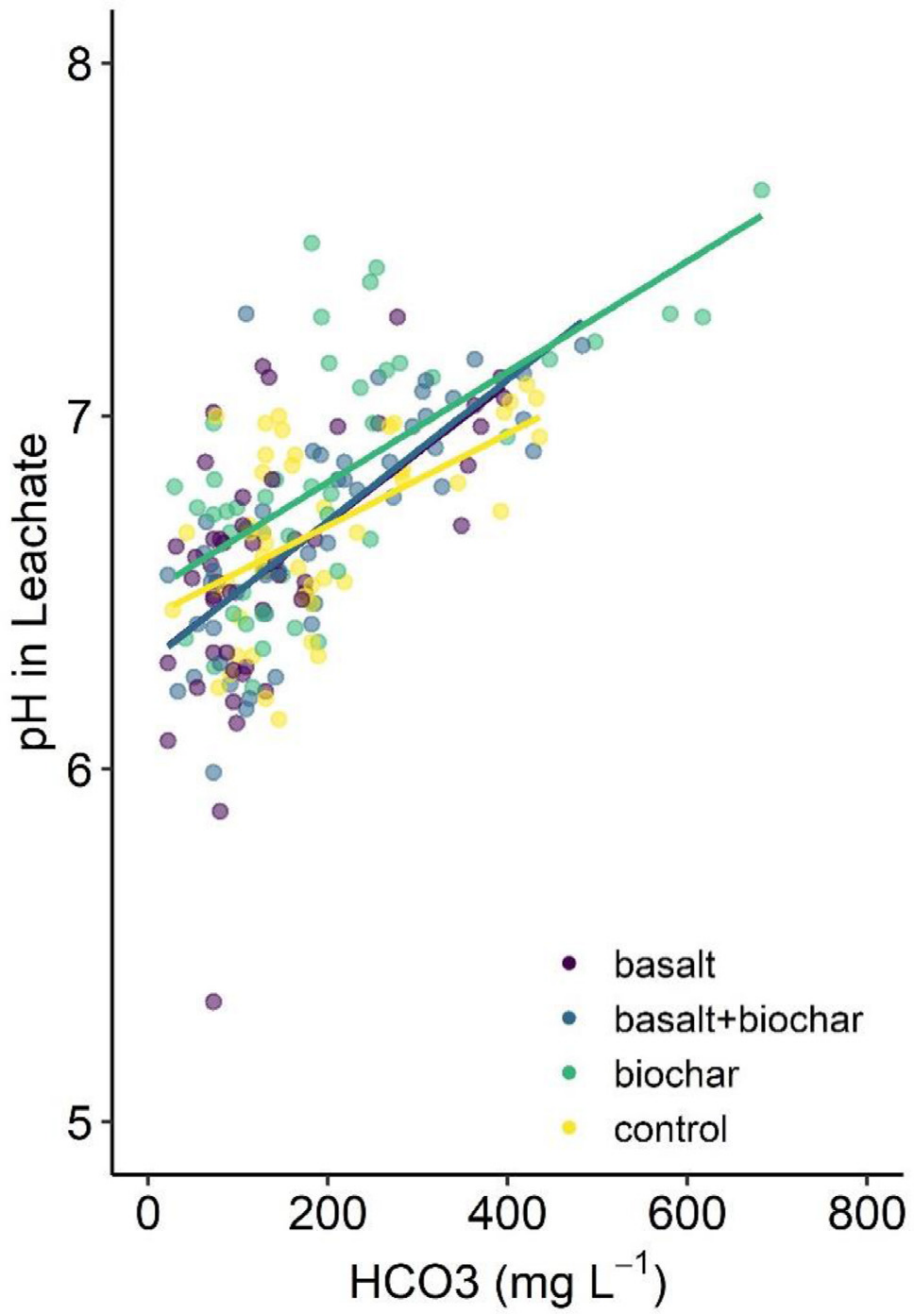
Relationship between pH in leachate and bicarbonate concentrations (mg l^-1^) for basalt and/or biochar treatments and unamended soil used as control.

The validation of the method is demonstrated by the efficient collection of the water leaching through the intact soil monoliths and accumulating at the system's base via the sampling tube, allowing for chemical analysis over a period of 6 months (since November 2023- April 2024); the experiment is continuing for 9 months. Despite the relatively short duration reported here, the preliminary results indicate that concentrations of bicarbonate (HCO_3_^-^), calcium (Ca^2+^), and magnesium (Mg^2+^) in leachates from treated soils increased relative to the control (Fig. 9). The highest increases were observed for the treatment combining basalt and biochar (see Fig. 10). Additionally, there is evidence that the increase in bicarbonate concentration is associated with an increase in pH (Fig. 11), which again helps to validate investigation of the weathering process by using the apparatus.

The elevated concentrations of HCO_3_^-^, Ca^2+^, and Mg^2+^ in leachates from treated soils, compared to the control, also suggest that water is infiltrating through the intact soil monolith rather than along the periphery of the pipe. This observation further validates the methodology for collecting and analysing water chemistry. However, it is important to note that these results are based on a specific soil type and location. While we strongly believe the method is applicable to various soil types and environmental conditions, results from other locations may differ.

Current research has emphasised the need for the development of such a methodology [7]. In this sense, our initial findings demonstrate the potential of the apparatus for making measurements necessary for validating models of CO_2_ removal using ERW methodologies (e.g. [4]). Accordingly, the method could facilitate a precise empirical assessment of ERW effectiveness in C removal under field conditions. Nevertheless, we stress the importance of fully understanding the holistic impact of this and other CDR approaches (e.g. biochar) in climate change mitigation. Beyond assessing impacts on water leachates, this method also offers insights into the effects on gas emissions from the soil and plant productivity, both of which can also be measured, and soil conditions, considering local variables and environmental factors, which should also be considered [8,9]. These aspects are highlighted in current publications, such as a recent review by Vicca et al. [8], which suggests that the presence of plant roots and microbial activity could significantly influence weathering rates and C capture efficiency. Additionally, another study [9] raised concerns about the potential accumulation of toxic trace elements from such practices, which again could be tested by using the method here proposed.

In conclusion, while this paper presents a validated method for collecting and analysing water leachates, further research using the method reported here is necessary to fully understand the broader implications of ERW and other CDR approaches. Addressing these broader scientific questions is beyond the scope of this methods paper and should be the aim of future publications.

## Limitations

In the course of the experiment, a few limitations, or potential areas for improvement, have become apparent, which could be easily addressed in future studies.

(1) In a few select locations, notably in soils with high clay and silt content, we encountered some difficulties when removing the PVC pipe from the ground. This was due to the pipe potentially lacking the necessary strength to extract the intact soil monolith without tearing the 20 mm holes located 25 mm below the top of the pipe. To address this issue, we inserted metal rings into the holes to provide additional support. Alternatively, another solution could be to increase the number of holes from two to four, thereby distributing force more evenly when extracting the intact soil monolith.
(2) While possible, the collection of leached water via the sampling tube was at times challenging because the basal storage chamber was air-tight: withdrawal of water would ultimately create a vacuum. A potential solution could be to insert an additional tube into the storage chamber to allow for the entry of air (or nitrogen from a gas-tight flexible bag) to compensate for removal of the water sample.
(3) Earthworms were discovered in some water samples. It is possible that the stainless steel mesh (grade 304, 5.1 mm aperture), the woven weed control mesh, and a geotextile membrane (100 µm mesh size) inserted into the middle of the double socket were insufficient to prevent earthworms from accessing and falling into the collected water. One potential solution could involve employing stainless mesh with a smaller aperture, effectively preventing earthworms (and potentially other invertebrates) from passing through while still permitting water flow.
(4) One further observation is not considered a limitation but rather a cautionary note. Depending on the soil's condition during extraction, the intact soil monoliths may undergo changes over time, such as shrinkage or expansion. This could potentially affect water flow through the soil. To mitigate this potential issue, we recommend collecting the soil monoliths during a period of the year when the soil moisture levels are neither excessively dry nor wet.
(5) Lastly, it is important to note that the results presented for the validation of the method are derived from a specific soil type at a particular location. Therefore, although we believe that the method is applicable to any soil type and environmental condition, the results used for validation may not be directly transferable to other locations.

## Ethics statements

The authors declare that the present method did not involve human subjects, animal experiments, or data collected from social media platforms and so ethics statements are not applicable.

## CRediT authorship contribution statement

**Caio F. Zani:** Conceptualization, Methodology, Writing – original draft, Writing – review & editing. **Arlete S. Barneze:** Conceptualization, Methodology, Writing – original draft, Writing – review & editing. **Gerlinde B. De Deyn:** Conceptualization, Methodology, Writing – review & editing. **J. Frans Bakker:** Conceptualization, Methodology. **Kevin Stott:** Methodology, Writing – review & editing. **David A.C. Manning:** Writing – review & editing, Funding acquisition.

## Declaration of competing interest

The authors declare that they have no known competing financial interests or personal relationships that could have appeared to influence the work reported in this paper.

## Data Availability

Data will be made available on request.
